# Conflicting Cultural and Religious Views on Cosmesis: The Modern Women’s Dilemma

**DOI:** 10.1007/s00266-022-02834-6

**Published:** 2022-04-06

**Authors:** Nada Raafat Khattab, Noha Abdelraouf, Tarek Ashour

**Affiliations:** Plastic Surgery Unit, Kasr Al Ainy Hospital, Cairo University, Al Manial, Cairo, 11562 Egypt

**Keywords:** BDD, Female gender role stress, Religion, Cosmetic procedures, Motivation, Health evaluation, Life satisfaction, TV exposure and social media

## Abstract

**Background:**

Women from countries with conflicting views on cosmesis may avoid these procedures for the fear of being rejected by the community. Understanding the motives that drive patients from these countries to seek cosmetic procedures helps discern possible causes of postoperative dissatisfaction, which can be prevented by careful selection of patients and individualizing their management protocols.

**Objectives:**

This study helps identify the factors that affect Egyptian Muslim women’s attitude toward cosmetic procedures. The main factors tested were female gender role stress (FGRS), body dysmorphic disorder (BDD), and religious attitude. The secondary factors investigated were health evaluation, life satisfaction, self-satisfaction, social media use, TV exposure, spouse/friends/family influence, and internalization of beauty standards.

**Methods:**

Women willing to undergo cosmetic procedures were compared with those who were not. A survey exploring demographics and the different motives were posted for the public online.

**Results:**

Among 502 participants, 288 were willing to undergo cosmetic procedures and 214 were not. Our findings showed a statistically significant difference for the degree of BDD, FGRS, and religiousness between willing and unwilling groups. Moreover, greater pressure from partner to change appearance, influence of friends and family on opinion regarding beauty of oneself, internalization of beauty standards, and lower ratings of life and self-satisfaction showed statistically significant association with willingness to undergo cosmetic treatment.

**Conclusion:**

BDD, FGRS, and religious attitude are among the highest predictors of the willingness of women to undergo cosmetic procedures together with many other factors. This study is the first of its kind to evaluate several unexplored motives and opens the door for future research.

**Level of Evidence V:**

This journal requires that authors assign a level of evidence to each article. For a full description of these Evidence-Based Medicine ratings, please refer to the Table of Contents or the online Instructions to Authors www.springer.com/00266.

## Introduction

"What is beautiful is good . . . [Sappho, Fragments, No, 101]."

Since the dawn of civilization, women have been seeking the “perfect” body. Ancient Egyptian women spent a lot of time in effort to look beautiful and retain their beauty [1]. Over 5000 years later, this seems to have not changed. Although beauty standards have varied across centuries, women’s immense desire to compel to these standards remains the same. This pattern of behavior has led women to severe measures not only in Egypt but around the world, including the Chinese habit of foot binding [2]. In the modern world, among the numerous methods of cosmesis, the most prominent are the procedures performed by cosmetic professionals.

Standards of beauty are believed to be dynamic and different from one culture to another. However, a meta-analysis argues that standards of attractiveness are pancultural [3]. Researchers explored the possible reasons behind the existence of beauty standards in a society and the sociocultural pressure on women to comply with them. They concluded that women that lie within these standards are viewed as possibly more fertile and elite [4, 5].

Mass media has revolutionized feminine beauty ideals. It is fair to say that women’s obsession over how they look increased dramatically with the increasing popularity of TV shows. Furthermore, the fashion industry encourages the irrational belief that beauty is that of the physical appearance only; it deceives women into thinking that there is a certain figure they “should” look like. Chronic exposure to content affirming to these ideals reinforces the internalization of beauty standards. It has reached a point that now people view beauty as a sign of goodness [6, 7].

In the literature, there are studies that encourage cosmetic surgery. They argue that for some women, their bodies may not represent their “real selves” and may make them look “alien and intrusive” [8, 9]. As supported by Davis, “Cosmetic surgery is about exercising power under conditions which are not of one’s own making. In a context of limited possibilities for action, cosmetic surgery can be a way for an individual woman to give shape to her life by reshaping her body. Cosmetic surgery is about morality. For a woman whose suffering has gone beyond a certain point, cosmetic surgery can become a matter of justice—the only fair thing to do” [8].

Consequently, women with insecurities and the urge to adhere to the culture standards are prone to develop psychological disorders such as body dysmorphic disorder (BDD). This psychological disorder, which is characterized by an unreasonable preoccupation with perceived defects in appearance, results in significant functional impairment [10]. It is associated with significant morbidity and mortality. For instance, susceptible people who are severely dissatisfied with how they look are more likely to develop depression, social anxiety, and obsessive compulsive disorder up to attempting suicide [11].

Women’s characteristics and motives to undergo cosmetic procedures have not been studied adequately. Previous studies that examined the factors that affect the likelihood of undergoing cosmetic procedures focused on demographics, self-esteem, media exposure, and psychological aspects. Very few studies if any considered factors such as female gender role stress (FGRS), religiousness, life satisfaction, self-satisfaction, health evaluation, internalization of beauty standards, and influence of close ones. To shed light on this, we have assessed these factors by using mainly Likert scales. We have also considered demographics, media exposure, and psychological aspects, namely body dysmorphic disorder, in order to establish comparisons with previous studies.

Indecisive women are on the horns of a dilemma. One element is whether to break cultural and religious views to follow the trend or fulfill a motive; the other is whether to indulge in cosmetic procedures and give themselves up to risks of these surgeries. There are indeed many motives that prompt women to opt for cosmetic procedures. On the contrary, there are factors that may discourage women to seek cosmetic procedures. These include opposing cultural and religious views. Among others, these two are very prominent in the Muslim Egyptian society [12, 13]. This has encouraged us to choose a sample form the Egyptian community, particularly Muslim Egyptian women.

The rationale of our study is that the sine qua non of obtaining a favorable outcome in the aesthetic practice is to determine the motivation underlying the patient’s interest in such procedures. Our objective is to provide substantial information that would aid in screening patients that are not suitable for cosmetic treatment.

## Methodology

**Fig. 1:**
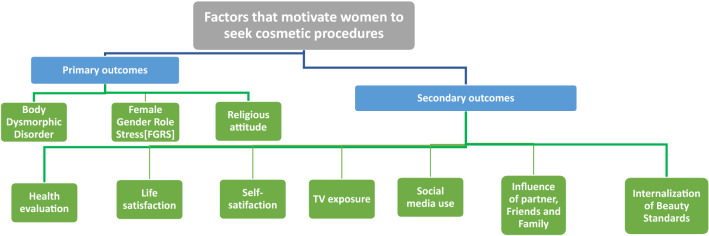
Outline of the factors that were investigated in the study

The objective of this study is to assess demographic characteristics and different motives of Muslim Egyptian women willing to undergo cosmetic procedures. We constructed an Internet survey (Appendix 1) to compare women who report willingness to undergo cosmetic procedures versus women who do not. We have investigated the significance of ten incriminated factors (Fig. 1).

The three main factors tested in our study, by validated Likert scales, were female gender role stress, body dysmorphia, and religious attitude. Religion was explored in our study as a primary outcome for its impact on well-being and therefore postoperative satisfaction [13]. The three validated Likert scales were translated by three translators into standard Arabic using the forward and backward translation procedure as recommended by Brislin [14]. The secondary factors we investigated were health evaluation, life satisfaction, self-satisfaction, social media use, TV exposure, spouse/friends/family influence, and internalization of beauty standards. The secondary outcomes were assessed using direct individual questions developed by the authors that were answered using Likert scales. Eight questions were used to collect information on the demographics.

The survey we conceived was uploaded online on Google Forms. There were Arabic and English versions. It was then made available to the public on three public groups/pages on Facebook targeting Egyptian women. The total followers of these platforms added up to approximately 1 million. The form was accessible the entire duration of the research from October 17, 2020, to March 16, 2021, and posted on the platforms three times. This is when we have also reached the number of participants that was deemed adequate.

On the consent page, the study objective was described as follows: “This questionnaire is for the purpose of assessing factors that motivate women willing to undergo any of the cosmetic procedures listed.” The participants were informed that their participation is voluntary, that there are no right and wrong answers, and that all responses are absolutely confidential and anonymous. They were also informed that the survey takes an average of 10 minutes to complete. Participants who agreed to participate were asked if they would be willing to undergo any cosmetic procedure if they were capable to do so. This was followed by the questions targeting the objectives of the questionnaire. Participants could not submit their response unless all questions were answered and were allowed to complete the survey only once.

## Sample Size

Consecutive sampling technique was used, and participants were recruited during a period of 6 months. In addition, to ensure optimal sample size, power analysis was performed using one of the primary outcomes, body dysmorphic disorder (BDD). Fleiss JL (1981) unequal sample size formula with continuity correction was used [15]. The estimated required sample size is 92 for willing participants and 99 for participants unwilling to undergo cosmetic procedures, which makes a total of 191. Adjustment for 80% response rate gives 239 total participants.

## Participants

The survey started by allowing only those who are in the inclusion criteria to proceed to the consent page and those in the exclusion criteria were asked to submit and exit the survey. The exclusion criteria were males, non-Egyptians, non-Muslims, and age less than 18 years. In addition, women who have undergone cosmetic procedures in the past were excluded. The reason behind this is that past experiences, positive or negative, may influence current willingness to undergo cosmetic procedures.

Out of the 804 participants, only 526 fulfilled the inclusion criteria and 502 agreed to participate. The response rate was 95%. Two hundred and eighty-eight were willing to undergo cosmetic procedures and 214 were not. Seventy-eight percent of the participants filled the survey in Arabic and the rest in English.

The usability and technical functionality had been tested before the distribution of the survey. At the outset of the study in July 2020, 3-month test–retest reliability was performed by administering the questionnaire to 19 participants; scores proved reliability of the survey. Cronbach’s alpha was used to measure internal consistency of the validated Likert scales of the primary outcomes, and a value of 0.7 or more was considered acceptable.

All analyses were performed using the Statistical Package for the Social Sciences, version 24 (SPSS Inc., Chicago, IL, USA). Results were considered statistically significant at *p *< 0.05. Median and interquartile range were utilized for quantitative variables, while frequency tables with percentages were utilized for qualitative variables and descriptive statistics. Chi-square test and cross-tabulations were done to analyze categorical variables. Meanwhile, Mann–Whitney test was used for quantitative data analysis. Correlations between ordinal variables were evaluated using Spearman’s rho correlation coefficient.

## Questionnaire

The questionnaire used is demonstrated in Appendix 1.

### Demographics

Participants were asked to provide demographic information that included age, weight, height, marital status, highest educational qualification, income status, exercise, and presence of psychiatric disorder.

### Primary Outcomes

#### Body Dysmorphic Disorder

The Dysmorphic Concern Questionnaire (DCQ) scale consists of seven questions developed by Oosthuizen et al. [16] to assess symptoms of body dysmorphic disorder (BDD). Response to each question is graded as “no concern (1),” “same as most other people (2),” “more than most other people (3),” or “much more than most other people (4)”; the sum is then calculated. The total score varies between 7 and 28. The higher the score, the more likely the patient suffers from body dysmorphic disorder symptoms and significant distress. Median and interquartile range were used to represent the data. Internal consistency for the present sample was adequate (Cronbach’s alpha = 0.85).

#### Female Gender Role Stress

Questions derived from female gender role stress (FGRS) scale developed by Gillespie et al [17] were used to assess the degree by which women experience stress related to stereotypical feminine gender role. The female gender role stress scales assess five main domains: (A) fear of unemotional relationships; (B) fear of physical unattractiveness; (C) fear of victimization; (D) fear of behaving assertively; and (E) fear of not being nurturant. The five questions were graded as 1 “I strongly disagree” to 5 “I strongly agree.” The total score varies between 5 and 25, and higher scores indicate higher female gender role stress and vice versa. Median and interquartile range were used to represent the data. Internal consistency for the present sample was adequate (Cronbach’s alpha = 0.74).

#### Religious Attitude

An eight-item scale developed by Ok et al [18] was used to assess the religious attitude of participants. In Ok et al.’s study, this scale was used to evaluate the religious attitude toward Islam. The questions were graded as 1 “I disagree strongly” to 5 “I agree strongly.” There were two questions that were negatively structured; in this case, “I strongly disagree” was given a score of 5 and “I strongly agree” was given 1. The total score varies between 8 and 40, and the higher the score, the more religious the participant. Median and interquartile range were used to represent the data. Internal consistency for the present sample was adequate (Cronbach’s alpha = 0.91).

### Secondary Outcomes

#### Health Evaluation, Life Satisfaction, Self-satisfaction, TV Exposure, and Social Media Use

These five unrelated individual variables were assessed by five directly asked questions (one question for each). For health evaluation, life satisfaction, and self-satisfaction, response was in the form of a scale ranging from 1 to 4. A score of 4 indicates health evaluation as excellent, life satisfaction and self-satisfaction as the highest, and vice versa. The questions were originally on a scale of 4, and in the analysis, we have converged 1 and 2 to represent a low score and 3 and 4 to represent a high score. TV exposure was assessed by the number of hours spent watching TV per week (< 3, 3–5, > 5). Social media use was determined by asking whether they use popular social media platforms regularly, and the choices were: yes, often, a little, and no. Internal consistency for the five questions is not relevant.

#### Spouse/Friends/Family Influence

This section consists of four unrelated individual questions. These consist of three directly asked questions about the influence of close ones on opinion and an additional question to determine the extent by which the participant has felt pressure to change her appearance by her partner. The response was in the form of a scale ranging from 1 to 4. A score of 4 indicates the highest level of influence/pressure. The questions were originally on a scale of 4 where 4 is the highest score, “totally agree,” and vice versa. In the analysis, we have converged 1 and 2 to represent a low score, “disagree,” and 3 and 4 represent a high score, “agree.” Internal consistency is not relevant**.**

#### Internalization of Beauty Standards

In our study, internalization of beauty standards was evaluated by assessing the agreeableness to four unrelated individual questions derived from sociocultural attitude toward appearance questionnaire (SATAQ-3) [19]. These questions were “I would like my body to look like the bodies of celebrities/models,” “I feel pressure from the media to change my appearance,” “Society influences my opinion on beauty standards,” and “Social media is an important source of information about fashion and beauty.” The statements were graded as 1 “strongly disagree” to 4 “strongly agree.” The higher the grade, the greater the acceptance or internalization of the prevailing sociocultural standards for appearance. The questions were originally on a scale of 4 where 4 is the highest score, “totally agree,” and vice versa. In the analysis, we have converged 1 and 2 to represent a low score, “disagree,” and 3 and 4 represent a high score, “agree.”

## Results

### Demographics

The demographic characteristics of our participants are presented in Table 1. There was no statistically significant difference between the two groups as regards all of the demographic variables.

**Table 1:** Demographic characteristics of participants willing to undergo cosmetic procedures versus those not willing

Demographic characteristics	Willingness		*P*-value
	Positive *N *= 288	Negative *N *= 214	
Age, median (IQR)	24 (22–29)	24 (22–28)	.859
BMI, median (IQR)	25 (23–29)	25 (22–28)	.473
*Marital status, N (%)*			
Single	189 (65.6)	135 (63.1)	.401
Engaged	18 (6.3)	16 (7.5)	
Married	72 (25.0)	61 (28.5)	
Widow	2 (0.7)	1 (0.5)	
Divorced	7 (2.4)	1 (0.5)	
*Highest educational attainment, N (%)*			
Secondary school	0 (0)	1 (0.5)	.506
High school	69 (24.0)	52 (24.3)	
University/institute graduate	219 (76.0)	161 (75.2)	
*Income in Egyptian pound, N (%)*			
Do not work	168 (58.3)	118 (55.1)	.854
< 3000	59 (20.5)	51 (23.8)	
3000–6000	32 (11.1)	26 (12.1)	
6000–10000	18 (6.3)	13 (6.1)	
> 10000	11 (3.8)	6 (2.8)	
*Perform regular exercise, N (%)*			
Yes	57 (19.8)	44 (20.6)	.832
No	231 (80.2)	170 (79.74)	
*Presence of psychiatric disorder, N (%)*			
Yes	68 (23.6)	38 (17.8)	.112
No	220 (76.4)	176 (82.2)	

### Primary Outcomes

#### Body Dysmorphic Disorder

**Fig. 2:**
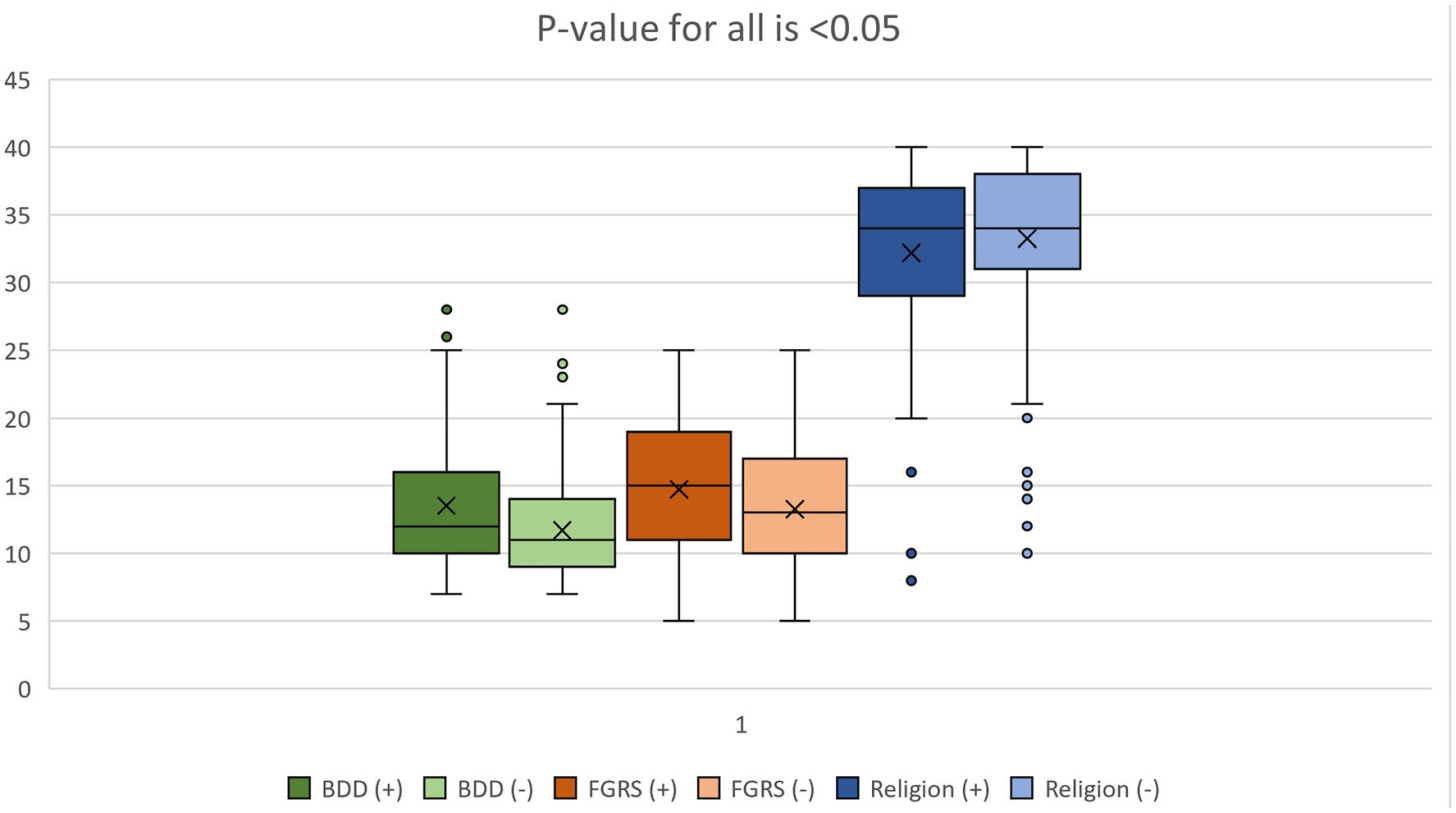
BDD, FGRS, and religiousness between willing and unwilling groups

The median body concern score is significantly greater in participants willing to undergo cosmetic procedures compared with those unwilling (*p*-value = 0.000) (Fig. 2).

#### Female Gender Role Stress

The median FGRS score is significantly greater in participants willing to undergo cosmetic procedures compared with those unwilling (*p*-value = 0.001) (Fig. 2).

#### Religious Attitude

There was a statistically significant difference in the religiousness between the willing and unwilling groups, *P*-value = 0.036 (Fig. 2). The group unwilling to undergo cosmetic procedures are more religious as evident by the higher religion scale scores.

### Secondary Outcomes

#### Health Evaluation, Life Satisfaction, Self-satisfaction, TV Exposure, and Social Media Use

There is no statistically significant difference between the two cohorts in health evaluation, TV exposure, and social media use. There is, however, a statistical significance in life satisfaction and self-satisfaction with *p*-value < 0.05. Increased willingness to undergo cosmetic treatment is associated with low life satisfaction and low self-satisfaction.

#### Spouse/Friends/Family Influence

There is no statistically significant difference between the two groups with respect to influence of partner on opinion. However, there are statistically significant positive relationships with respect to the other variables: pressure from partner to change appearance, influence of friends on opinion, and influence of family on opinion, all with a *p*-value = 0.05. High pressure from partner to change appearance as well as influence of family and friends on opinion is associated with increased willingness to undergo cosmetic treatment.

#### Internalization of Beauty Standards

All four questions assessing the internalization of beauty standards between participants were statistically significant (*p*-value < 0.05) (Table 2). Women willing to undergo cosmetic procedures showed higher levels of internalization of beauty standards.

**Table 2:** Internalization of beauty standards questions (1 and 2 = disagree, 3 and 4 = agree)

		Willingness			*P*-value
		Positive (%) *N *= 288	Negative (%) *N *= 214		
I would like my body to look like the bodies of celebrities/models	1,2	135 (46.9)		128 (59.8)	0.004
	3,4	153 (53.1)		86 (40.2)	
I felt pressure from the media to change my appearance.	1,2	163 (56.6)		148 (69.2)	0.004
	3,4	125 (43.4)		66 (30.8)	
Society influences my opinion on beauty standards.	1,2	148 (51.4)		145 (67.8)	0.000
	3,4	140 (48.6)		69 (32.2)	
Social media is an important source of information about fashion and beauty	1,2	45 (15.6)		52 (24.3)	0.015
	3,4	243 (84.4)		162 (75.7)	

### Comparisons Involving the Primary Outcomes

The primary outcomes were compared between different groups to elicit statistical significance (Table 3). For BDD, it is significantly more common among obese (BMI of 30 or more) participants, as well as those who are not in a relationship and those who suffer from a psychiatric disease. As for FGRS, it is significantly more likely to be reported in single participants and among those who do not work. When taking income into consideration, higher FGRS scores were found in those earning less than 6000 Egyptian pounds per month.

**Table 3:** Comparisons involving primary outcomes

	BDD median (IQR)	*P*-value
*BMI (N)*		
< 30 (412)	11.0 (9–15)	0.032
≥ 30 (90)	13.0 (10–16)	
*Presence of psychiatric disease (N)*		
Yes (106)	14.0 (10–18)	0.000
No (396)	11.0 (9–14)	
*Marital status (N)*		
Single (324)	12.0 (10–16)	0.000
Married (133)	10.0 (8–13)	

	FGRS median (IQR)	*P*-value
*Marital status (N)*		
Single (324)	15.0 (12–19)	0.000
Married (133)	11.0 (7–15)	
*Work (N)*		
Yes (216)	13.0 (9–18)	0.025
No (286)	15.0 (11–18)	
*Income in Egyptian pound (N)*		
< 6000 (168)	14.0 (10–18)	0.011
> 6000 (48)	10.0 (7–18)	

Analysis of the following ordinal data: FGRS, BDD, religious attitude, health evaluation, life satisfaction, and TV exposure, was done using Spearman’s rank correlation coefficient (Table 4). There are statistically significant positive correlations between (1) FGRS and BDD, (2) FGRS and TV exposure, (3) BDD and TV exposure, (4) religious attitude and life satisfaction, and (5) religious attitude and health evaluation. There are statistically significant negative correlations between (1) FGRS and life satisfaction, (2) BDD and health evaluation, (3) BDD and life satisfaction, (4) BDD and religious attitude, and (5) religious attitude and TV exposure.

**Table 4:** Spearman’s correlation of FGRS, BDD, TV exposure, health evaluation, life satisfaction, and religious attitude across our sample

		FGRS	BDD	TV exposure	Health evaluation	Life satisfaction	Religious attitude
Spearman’s rho	FGRS						
	BDD	.435^**^					
	TV exposure	.181^**^	.135^**^				
	Health evaluation	− .005	− .090^*^	− .037			
	Life satisfaction	− .263^**^	− .308^**^	− .059	.165^**^		
	Religious attitude	.045	− .171^**^	− .133^**^	.138^**^	.287^**^	

**. Correlation is significant at the 0.01 level

*. Correlation is significant at the 0.05 level

## Discussion

Our findings showed that most of our respondents were single, Muslim, overweight young adults who have at least a bachelor’s degree but do not work. Only a small percentage of our participants exercise regularly and/or are diagnosed with a psychiatric disease. In comparison with other papers investigating demographics of cosmetic patients, these papers showed that the average female willing to undergo a cosmetic procedure is married and employed with a high monthly income [20, 21]. This may be explained by the fact that our study explores the theoretical interest in surgery rather than the actual pursuit.

There are several papers that support our finding that cosmetic patients have greater levels of body image dissatisfaction [22–24]. The fact that society stereotypes obese people as unattractive, less competent, lazy, and older could explain why in our sample individuals with BMI of 30 or more had greater dysmorphic concern [25, 26].

In this study, we found that BDD symptoms are greater in individuals with lower life satisfaction, health evaluation, and religiousness (less spiritual). BDD being higher among those with less spirituality is supported by a study exploring intrinsic religious orientation and body dissatisfaction [27]. This could be explained by the harsher evaluation of one’s body in these circumstances.

Our findings established a positive correlation between BDD and TV exposure. Hence, in this study the internalization of beauty standards is associated with higher willingness to undergo a cosmetic procedure. However, our findings reveal that TV exposure on its own does not predict willingness to undergo cosmetic treatment. This is supported by a study investigating TV exposure, especially cosmetic surgery shows, on teenage girls. Exposure to these shows resulted in more body image dissatisfaction, but no changes were observed in attitudes toward cosmetic surgery [28].

Our study also revealed a statistical significance of BDD being closely related to other psychiatric disorders. This is consistent with several other studies [29–31]. Two of these studies also affirmed that patients with BDD were more likely to experience more severe psychiatric symptoms than patients not suffering from BDD [29, 30]. In contrast to the results of Conroy et al., our study revealed that being single was associated with BDD. This result may be due to cultural differences between the Egyptian culture and that of the west [30].

The virtue behind the domains explored by the FGRS is to investigate whether women experience stress under certain circumstance if they do not comply with female gender role imperatives [17]. Our findings established a correlation between female gender role stress and women who do not work and whose income was less than 6000 EGP. It is not clear whether the low income or lack thereof harbored the stress or the opposite, but it may be because groups of people who earn lower incomes are more prone to societal stress in general for their inability to provide for themselves independently. FGRS was also correlated with women being single. This area of research is unfathomed in Egypt and other Arab communities and therefore requires further research.

Although most cosmetic procedures are gender neutral, experiences of embodiment between men and women suggest that cosmetic surgery has very different meanings for the two genders [32]. Women reported higher degrees of body dissatisfaction compared to men [33]. However, the “threatened masculinity” hypothesis postulates that societal changes led to a significant increase in men’s muscularity dissatisfaction [34]. Furthermore, equality discourse which currently pervades modern culture neutralizes the salience of gender [32]. In a study conducted on men of similar populace and criteria, men seeking cosmetic treatment reported high rates of gender role stress [35]. Our study has revealed similar findings in terms of gender stress and willingness to undergo cosmetic procedures. To understand whether a difference truly exists between male and female gender role stress in cosmetic willingness, we encourage further research to compare the two genders in terms of willingness to undergo cosmetic surgery.

“Body modification practices are generally elective, and tend to be driven by aesthetic, cultural, religious, or symbolic considerations”—Lauren Elliott [36]. To many people, religion guides the disciplines to be followed and therefore the attitude toward many controversial topics. Whenever religious beliefs are brought up in the field of aesthetics, the proper management is always in question. It is crucial to remain on the same page with patients, regardless of their faith, because spiritual well-being is an important component of health.

Our sample consists of Muslim women. The stance of the literature with respect to Islam and Aesthetic surgery tends to discourage undergoing such procedures [13]. There was a statistically significant difference in religiousness between the willing and unwilling cohorts in our study. However, a paper on men, all of whom were Muslims, who sought cosmetic treatment revealed no statistically significant difference between religiousness and cosmetic attitude [35]. A study on Christianity revealed that the more religious a person is the higher the probability that they would view cosmetic treatment as a violation to their beliefs. To our knowledge, there are no other papers that examine religion’s effect on the willingness to undergo cosmetic treatment [37]. Our findings on religious attitude and willingness to undergo cosmetic treatment warrants further research to involve different faiths on a large scale.

However, in the literature, there are studies that investigate the debate of religion, especially the three main Abrahamic religions (Judaism, Christianity, and Islam), and cosmetic procedures. The rule in Islam is that individuals should be satisfied with the way God has created them [13, 38]. As for Christianity, it is recommended that individuals be more focused on religious issues than appearance, arguing that beauty lies within the spirit [37]. In Judaism, Snyderman noted that it is permissible for Jewish individuals to undergo cosmetic treatment, arguing that Judaism honors beautifying oneself and the procedure is unlikely to be harmful [39]. However, there are papers that question the previous statement arguing with evidence that it is prohibited unless correcting congenital or acquired deformities [40–42]. It is clearly a controversial topic among Jewish patients with different teachings. In general, it is difficult to assume that people belonging to a specific religion have homologous ideologies; there are different cultures and schools within each religion. All in all, the vast majority of studies on the three religions agree that there should be a real medical indication to justify the cosmetic procedure [38, 43]. The discrepancy of the religious authorities point of views with the rising demands for cosmetic treatment warrants further research on the attitudes toward cosmetic procedures of individuals with different religious beliefs.

Our findings showed that higher BDD, FGRS, and religiousness are significant predictors of the willingness to undergo cosmetic surgery. Moreover, greater pressure from partner to change appearance, influence of friends and family on opinion in self-beauty, internalization of beauty standards, and lower ratings of life satisfaction and self-satisfaction were all significantly associated with willingness to undergo cosmetic treatment. On the other hand, despite that someone would expect that the time spent watching television, social media use, health evaluation, and influence of partner on opinion would affect the likelihood of women to undergo cosmetic procedures, our study does not prove such correlations. Those are important findings, especially for media exposure, as this was concluded by a systemic review as well [44].

This study provides insights on the motives of women willing to undergo cosmetic procedures. Our outcomes offer a clearer understanding of the various factors that affect a woman’s decision to undergo a cosmetic treatment and therefore should be taken into account by cosmetic professionals when considering how and when to treat patients seeking cosmetic treatment.

## Strengths and Limitations

This study is the first of its kind to evaluate unexplored variables such as FGRS as motives for desiring cosmetic procedures, which opens the door for further research. There are limited prior research studies on the topic, especially for certain factors we investigated. This has shed light on the gaps and limitations in the finite preexisting literature that emphasized the need to be explored. However, it is not without limitations. Given that our study is conducted via the Internet, it may not represent the demographics of the actual population presenting in cosmetic clinics. Although validated scales are available for internalization of beauty standards, the number of questions in the validated scales was huge to fit into our already long questionnaire. Therefore, we used only a few questions to represent these aspects to point out any possible significance that could be studied in further research with the validated questionnaire.

## Conclusion

There are diverse reasons for the dissatisfaction of patients with the results of cosmetic procedures. Since prevention is the best treatment of a dissatisfied patient, it is important to identify the motives that drive the desire for these procedures preoperatively. This study established that high degrees of BDD, FGRS, and religiousness do indeed have a significant effect on the willingness to undergo cosmetic procedures. Moreover, our study proved significance of other factors that could motivate women to seek cosmetic procedures. Therefore, it is hoped that this project will be the beginning of an ongoing body of research to broaden the knowledge on the different aspects that affect patients’ postoperative satisfaction.
